# Spontaneous Progressive Muscle Weakness with Persistent Leukocytosis

**DOI:** 10.1155/2019/7085219

**Published:** 2019-07-09

**Authors:** Sameer A. Hirji, Manish M. Karamchandani, Jonathan W. Scott, Matthew T. Menard

**Affiliations:** Department of Surgery, Brigham and Women's Hospital, Harvard Medical School, Boston, MA, USA

## Abstract

Iliacus compartment syndrome is a rare clinical condition which can result in a severe, unilateral, femoral neuropraxia. Recognition of this syndrome as the cause of a developing neuropathy is often delayed given a lack of familiarity with this clinical diagnosis and the retroperitoneal location of the iliacus muscle. Prompt diagnosis is important to avoid risk of consequent muscle necrosis, rhabdomyolysis, and possibly permanent nerve injury. We describe a case of iliacus compartment syndrome in an elderly, frail woman with end-stage renal disease, anticoagulated for atrial fibrillation, who presented with subacute, progressive lower extremity muscle weakness and pain in the setting of complicated metabolic derangements. She was found to have a spontaneous large hematoma in her left iliacus muscle on computed tomography scan. Despite an initial diagnostic delay, she was successfully managed with an iliacus fasciotomy, which led to complete resolution of her symptoms.

## 1. Introduction

Our case demonstrates a rare but often overlooked compartment neuropathy: iliacus compartment syndrome. We highlight the importance of considering iliacus, iliopsoas, and pelvic compartment syndromes in the differential diagnosis during a workup of lower extremity pain and muscle weakness, particularly in elderly and frail patients and those on anticoagulation. Due to the deep retroperitoneal location of the iliacus muscle, pathology arising from a spontaneous bleed within the iliacus fascial sheath has few localizing signs and symptoms to help pinpoint the etiology of more distal symptomatic manifestations; this stands in contrast, e.g., to a counterpart bleeding problem, that of a rectus sheath hematoma, where a developing thrombotic mass can often be appreciated visually or by palpation. Additionally, given the nonspecific and often subacute presentation, and the typical absence of a triggering event, there can be a significant delay in diagnosis and management of iliacus compartment syndrome, with the potential for neurologic sequela that can begin insidiously but progress to the point of irreversibility. The presence of baseline neurologic, vascular, or orthopaedic comorbidities in the patient population at higher risk for this clinical problem can further contribute to diagnostic confusion, adding to the difficulty in discerning the origin of lower extremity nerve deficits and pain that are characteristic of its later stages. If undetected and untreated, patients with iliacus and pelvic compartment syndromes can develop progressive muscle necrosis, rhabdomyolysis, and acute kidney injury [1, 2]. For these reasons, it is necessary to be aware of this uncommon pelvic compartment syndrome, particularly in hospitalized and critically ill patients in which the diagnosis can be masked by other attendant medical issues.

## 2. Case Presentation

A 73-year-old frail woman with extensive comorbidities, including rheumatic heart disease (status post mechanical aortic and mitral valve replacement for which she was on warfarin therapy), nonischemic cardiomyopathy and chronic atrial fibrillation (status post implantable cardiac resynchronization therapy), a history of cerebral haemorrhage, type II diabetes mellitus, chronic kidney disease, congestive heart failure, and peripheral artery disease, presented with a 1-month history of progressive, debilitating bilateral thigh muscle weakness (left more than right) and left lower extremity pain, which caused difficulty in walking and arising from a seated position. She also endorsed intermittent symptoms of bilateral lower extremity rest pain and claudication. The patient remained hemodynamically stable and afebrile throughout her hospital stay. On physical examination, she had significant muscle atrophy (bilateral thigh and calf muscles) and reproducible pain with active and passive movements of her left hip muscles. Her calf and leg compartments were soft bilaterally, although with focal tenderness along the left lateral thigh. A lesser degree of tenderness was present on the right lateral thigh. Her sensory exam was limited due to her baseline diabetic neuropathy and peripheral artery disease.

The patient underwent an extensive diagnostic workup, which demonstrated significant metabolic derangements of unclear etiology. Notably, her relevant laboratory abnormalities included persistent leukocytosis in the 19,000 white blood cell/mcL range, rise in her creatinine from 1.1 to 1.4 mg/dL, potassium of 4.7 mEq/L, lactate of 1.9 mg/dL, creatinine kinase of >1000 U/L, an erythrocyte sedimentation rate of 80 mm/hr, C-reactive protein of 117 mg/L, and procalcitonin of 0.28 ng/mL.

The neurology service was initially consulted to assess her bilateral lower extremity pain and weakness and concluded that her clinical picture was inconsistent with a neuropathic process. Given a family history of systemic lupus erythematosus, the rheumatology service was then consulted to assess for the possibility of inflammatory myositis or vasculitis. Their initial impression was that of possible inflammatory myositis, and they recommended a muscle tissue biopsy for further evaluation.

**Figure 1:**
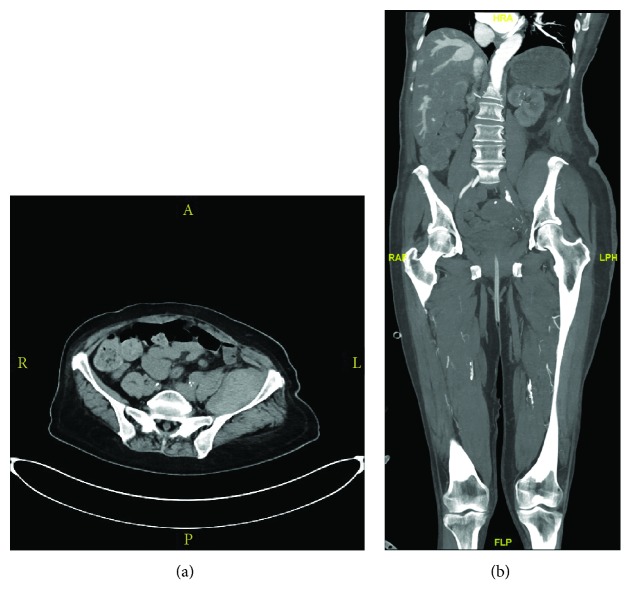
Contrast-enhanced computed tomography of the abdomen and pelvis showing a large intramuscular hematoma of the left iliacus muscle which measures 7.6 × 3.9 cm without active extravasation: (a) axial image and (b) coronal image.

With the diagnosis still unclear, the emergency surgery service was next consulted to rule out lower extremity compartment syndrome; suspicion for this was low in the presence of soft calf and leg compartments. At this point, the patient underwent abdominal and pelvic CT angiography (CTA), which demonstrated a 7.6 × 3.9 cm intramuscular hematoma within the left iliacus muscle, without evidence of active extravasation (Figures 1(a) and 1(b)). Also noted were severe, diffuse multifocal atherosclerotic plaque throughout the abdominal aorta and iliac arteries, an ostial occlusion of the right superficial femoral artery (SFA), and a midsegment left SFA occlusion. Her ankle brachial indices were determined to be 0.55 on the right and 0.53 on the left, consistent with aortobi-iliac and bilateral femoral artery occlusive disease. The vascular surgery service was then consulted due to these findings and persistent diagnostic confusion. After consideration of the patient's progressive and ongoing symptoms, imaging findings, and history of anticoagulation use, she was diagnosed with iliacus compartment syndrome, a rare and underappreciated entity, causing femoral nerve ischemia in the setting of large iliac sheath hematoma.

Given ongoing and progressive symptoms of unilateral left lower leg weakness and pain, the patient was taken urgently for open surgical retroperitoneal exploration. The left iliac sheath was exposed after developing the retroperitoneal plane through a modified left flank (Gibson's) incision. The iliacus fascia was found to be tensely expanded on inspection and palpation. A fasciotomy was carried out by sharply incising the fascia in both a cranial and caudal direction, and approximately 500 cc of dark-coloured, gelatinous thrombus was evacuated. No specific site of bleeding was identified during surgery (nor on preoperative CTA). The evacuated hematoma space was copiously irrigated, and drains were placed in both the subfascial cavity and the left lateral retroperitoneal gutter.

The operative procedure and postoperative course were uncomplicated. The patient reported early improvement in leg pain on postoperative day 1 and slow return of left leg hip flexion motor function by postoperative day 2. Given her complicated medical history, she remained on the cardiology service for 2 additional weeks for medical management of her congestive heart failure and optimization of her anticoagulation. She continued to work with physical therapy while in-hospital and was eventually discharged to a rehabilitation facility for further recovery. At discharge, she was doing well and with significant improvement of her left leg motor function.

## 3. Discussion

Iliacus (or iliopsoas) and pelvic compartment syndromes are rare compartment neuropathies characterized by increased intracompartmental pressure, potential compression of the femoral nerve, and varying degrees of resultant neurologic dysfunction. The increased intracompartmental pressure can be caused by bleeding from blunt trauma [3], prolonged stasis secondary to medication overdose, or an iatrogenic femoral or iliac vessel injury. Hematomas in the retroperitoneal location can also occur spontaneously in patients on anticoagulant medication. Bosch and Tscherne [4] described a typical case of a 52-year-old man on oral anticoagulation after a mechanical aortic valve replacement who developed a large gluteal hematoma over the week following a fall. Mwipatayi et al. [5] described a case of a 75-year-old elderly man on warfarin anticoagulation who presented with a 2-week history of hip and leg pain and unilateral femoral nerve distribution leg weakness (i.e., decreased proximal hip flexion with diminished power in the L3-S1 myotomes) after a recent attempted balloon aortic valvuloplasty.

A spontaneous iliacus hematoma in the absence of prior trauma or iatrogenic vessel injury, as was seen in this patient, is an uncommon clinical occurrence. This case illustrates the danger of a delay in diagnosis and surgical treatment, given the risk of irreversible nerve injury and consequent permanent motor impairment and gait abnormalities. Particularly unique features of this case include the prolonged 4-week delay prior to presentation, the extent of her metabolic disarray, and additive confusion caused by the presence of lower extremity symptoms contralateral to the side of the causative hematoma. Her clinical status during her initial hospital course was further exacerbated by her existing comorbidities and suboptimal nutritional status.

Diagnosis and management of compartment syndrome, especially involving the iliacus and psoas muscle, are challenging and often delayed. The most common reason for such delay is lack of awareness of this clinical entity and a failure to match the symptoms with the offending source of nerve compression. This diagnostic dilemma, in part, can and should be resolved with thorough neurologic examination, which should be done serially to assess for progressive symptomatology, and prompt tomographic imaging when the index of suspicion is appropriately raised. The ultimate treatment strategy involves surgical decompression of the compartments with a fasciotomy, which serves both confirmatory diagnostic and therapeutic purposes. An important teaching point is that surgical decompression should almost always be offered and typically be withheld only for patients too unstable to undergo an operation, regardless of the duration of time to diagnosis and of the neurologic compromise, given the high degree of unpredictability with regard to recovery of peripheral motor and sensory nerve functions.

## 4. Learning Points

- Iliac muscle compartment syndrome is an uncommon entity but one with potentially dire consequences if undiagnosed and untreated
- The diagnosis of iliac muscle compartment syndrome is often delayed, given poor awareness of its existence. It should be considered in the differential diagnosis during the workup of unilateral lower extremity weakness and pain, particularly in elderly, anticoagulated patients
- While intramuscular hematomas can occur in the setting of blunt trauma, supratherapeutic anticoagulation status, or iatrogenic causes, spontaneous hematomas occur on a regular basis and can result in various compartment syndromes. The diagnosis of iliacus compartment syndrome can be facilitated by CTA or magnetic resonance imaging of the abdomen and pelvis, the findings of which should be correlated with appropriate clinical signs and symptoms
- Definitive management involves surgical decompression of the iliacus or iliopsoas compartmental fascia, which can be readily achieved via a retroperitoneal approach
